# Purely venous compression in trigeminal neuralgia—can we predict the outcome of surgery

**DOI:** 10.1007/s00701-022-05176-z

**Published:** 2022-03-11

**Authors:** Jörg Baldauf, Ehab El Refaee, Sascha Marx, Marc Matthes, Steffen Fleck, Henry W. S. Schroeder

**Affiliations:** Department of Neurosurgery, University Medicine Greifswald, Greifswald, Germany

**Keywords:** Trigeminal neuralgia, Venous compression, Microvascular decompression, Vascular compression syndromes

## Abstract

**Purpose:**

Controversies regarding venous compression and trigeminal neuralgia (TN) still exist. The study demonstrates our experience for microvascular decompression (MVD) in TN caused by purely venous compression. The goal was to identify prognostic anatomical or surgical factors that may influence the outcome.

**Methods:**

Between 2004 and 2020, 49 patients were operated with purely venous compression. Average age was 58.4 years. Mean history of TN was 7.8 years. Microsurgical procedures included transposition or separation of the vein, coagulation, and division. Several features have been analyzed with respect to BNI scores.

**Results:**

Evaluation on discharge revealed a complete pain relief in 39 (80%), partial improvement in 7 (14%), and no benefit in 3 (6%) patients. Facial hypesthesia was reported by 14 (28.6%) patients. Mean follow-up (FU) was 42.1 months. BNI pain intensity score on FU revealed 71.4% excellent to very good scores (score 1: 32 (65.3%); 2: 3 (6.1%)). BNI facial numbness score 2 could be detected in 13 patients (26.5%) during FU. There was no statistical relationship between immediate pain improvement or BNI pain intensity score on FU with respect to surgical procedure, size of trigeminal cistern, type of venous compression, venous caliber, trigeminal nerve indentation, or neurovascular adherence. BNI facial numbness score was dependent on type of venous compression (*p* < 0.05).

**Conclusion:**

We did not find typical anatomical features that could either predict or influence the outcome regarding pain improvement or resolution in any form. Neither classic microvascular decompression (interposition/transposition) nor sacrificing the offending vein made any difference in outcome.

## Introduction

Vascular compression of the trigeminal nerve root by an arterial loop at the root entry zone (TREZ) as the main cause of trigeminal neuralgia (TN) is most frequent. But a neurovascular conflict between the trigeminal nerve and a vein can be an acceptable cause of TN too. Barker et al. reported in 1996 the high number of 151 patients in whom a vein only was expected to be responsible for TN [1]. Besides their good results, they and others mentioned a high rate of recurrences of TN after microvascular decompression (MVD) of venous origin [12, 19]. A combination of an arterial and venous compression in TN is frequently seen, but the main offending vessel cannot always be determined [1, 17, 21, 22]. Dumot et al. reported no difference in outcome between patients with purely venous compression or with marked venous compression accompanied by an artery [2]. Several anatomical and clinical studies have focused on the characterization of veins related to the trigeminal nerve [3, 6, 7, 11, 16]. With respect to the venous anatomy different options for MVD are proposed, including coagulation/division or transposition of a vein. The surgical options regarding veins are different to arteries because of its morphology. Arteries can be mobilized from the nerve more easily compared to veins. It is because of their thick and stable wall and presence of clear defined arachnoid adhesions. In contrast, the venous wall is thin, vulnerable and frequently fixed to the nerve without a good arachnoid plane. That makes manipulation, transposition, or interposition more difficult [7]. Secondly, coagulation/division of veins increases the risk of venous congestion, cerebellar or brainstem edema or bleeding respectively [7, 22]. There is a small number of studies dealing with pure venous compression in TN [2, 4, 6, 7, 9–11].

We demonstrate our experience using the endoscope-assisted technique for MVD in patients with TN caused by purely venous compression. The goal of the study was to answer the question whether there are prognostic anatomical or surgical factors that may influence the outcome for these patients.

## Patients and methods

We reviewed our prospectively maintained data base of 262 patients with TN treated between 2004 and 2020. Only patients with a venous compression were included in the study. Patients with a neurovascular conflict not only related to a vein but also to an artery were excluded. 49 (18.7%) patients were identified and verified by operating charts, intraoperative images or videos respectively (Table 1). All patients received medical therapy before surgery either with poor benefit or appearance of undesirable side effects. The average age was 58.4 years (range: 20–86 years). There were 34 female and 15 male patients. The mean history of TN was 7.8 years (range: 1–22 years). All patients suffered from unilateral pain that was typical in 39 and atypical (permanent subliminal pain in combination with typical attacks) in 10 patients. The pain was mainly located in combination of territories V2/V3 (16 patients), followed by V3 only (14 patients), and V2 (11 patients) respectively. A mixture of territories V1/V2 was reported by 7 patients and all territories were affected in one patient.

**Table1:** Demographic data

Number of patients	49
Age (years)	58.4 (range 20–86)
Sex	
Male Female	15 34
TN	
typical atypical	39 10
Duration of Symptoms (years)	7.8 (1–22)
Side	
left right	20 29
Branches involved	
V2 V3 V1 + V2 V2 + V3 V1-3	11 14 7 16 1

## Surgical technique

Patients were positioned supine with a moderate elevation of the head and upper body. The head was rotated to the opposite side.

A superior retrosigmoid approach was performed and the endoscope-assisted microsurgical technique was used in all patients. Most surgical steps and dissection procedures were done under microscopic view. The endoscope was applied for primary free-hand inspection of the trigeminal nerve and offending vein before MVD. The local neurovascular anatomy was examined and compression site confirmed.

**Fig. 1:**
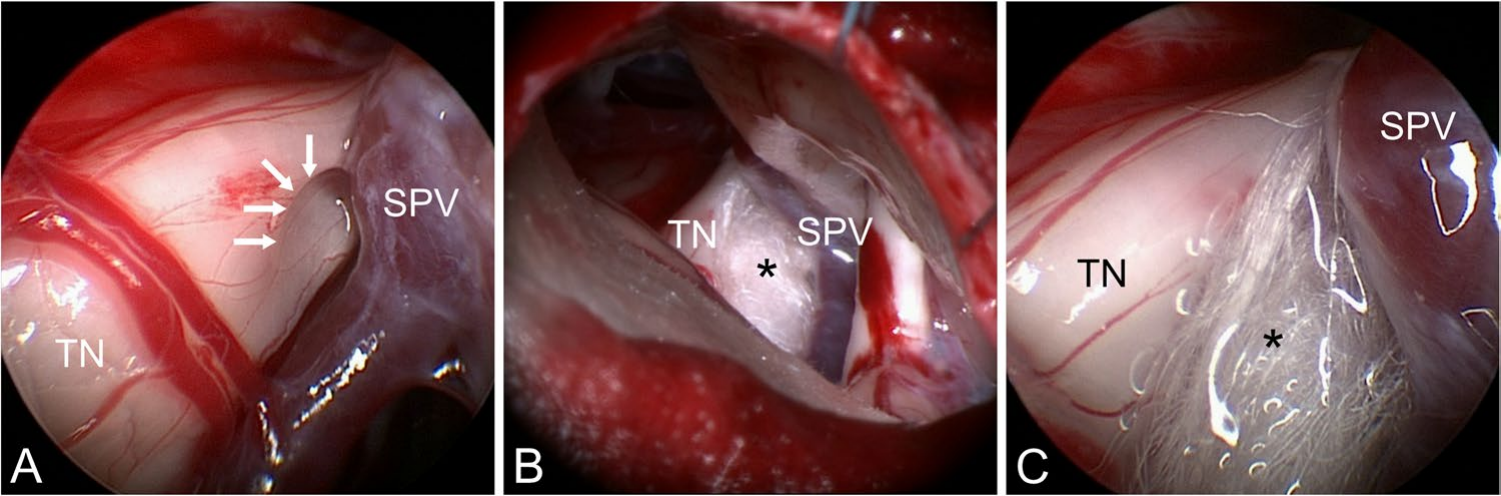
Typical venous compression (right side) resolved by microvascular decompression. A) Endoscopic view: superior petrosal vein (SPV) is separated from the trigeminal nerve (TN). Indentation to the nerve is impressively shown (white arrows). B) Microscopic view: Teflon sponge (asterix) is placed between TN and SPV. Tiny transverse crossing venous branches have been coagulated and divided. C) Endoscopic view after decompression

**Fig. 2:**
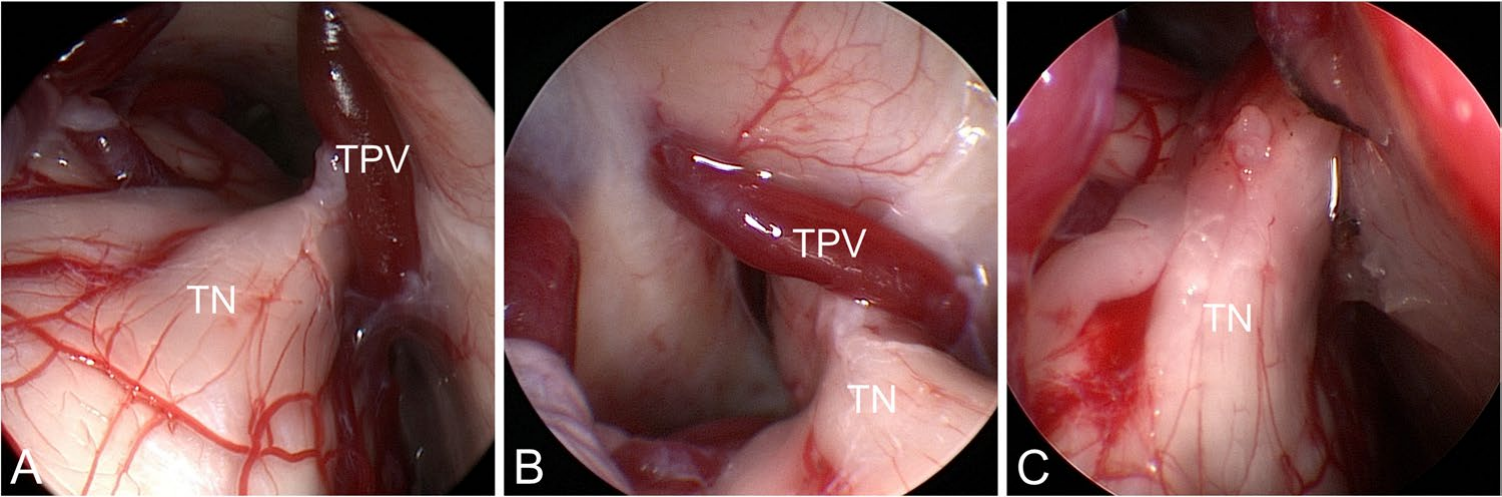
Transverse petrosal vein (TPV) crossing the trigeminal nerve (TN) close to Meckel’s cave (endoscopic images A + B). C) TN after coagulation and division of the TPV

Microsurgical procedures included transposition of the vein from the trigeminal nerve with insertion of a small piece of shredded Teflon between nerve and vein (Fig. 1) or separation of the vein, coagulation and division (Fig. 2). Coagulation/division was dependent on size of the offending vein and presence of collaterals. Only small veins were sacrificed.

Recording of brainstem auditory evoked potentials (AEP) and facial electromyography monitoring was obligatory in every patient.

## Anatomical features obtained intraoperatively

Several anatomical features had been documented during surgery. The vein and its relation to the trigeminal nerve was classified according to the representative types of TN by venous compression (Table 2) described by Inoue et al. [7]. The caliber of the vein was evaluated in relation to a 2-mm suction tip. Calibers were differentiated in small (< 2 mm), medium (2 mm), and large (> 2 mm). The size of the trigeminal cistern was measured on high-resolution T2-weighted axial CISS MR images. Cross-sectional measurement of the cisternal area and cisternal length of the trigeminal nerve was performed according to Park et al. and Pang et. al [14, 15]. A mean value was calculated. For analysis, the trigeminal cisterns were divided in narrow (< mean value) and wide (< mean value).

**Table 2:** Type of venous compression by Inoue et al. (2017)^7^

Type	
A	a small TPV joins the SPV or the VCPF after neurovascular compression at the mid-cisternal portion or root entry zone of the trigeminal nerve. The TPV may pass on either side of the nerve
B	a small TPV enters the SPS directly, compressing the nerve around the porus
C	a large TPV enters the SPS directly, compressing the nerve around the porus
D	the SPVor VCPF adheres and compresses along the nerve

*TPV* transverse pontine vein, *SPV* superior petrosal vein, *SPS* superior petrosal sinus, *VCPF* vein of the cerebellopontine fissure

With respect to our experience according to the adherence of vein and trigeminal nerve, we distinguished two types. Type 1, where an obvious arachnoid layer between nerve and vein appeared to be very thin or was missing. Type 2 describes nice anatomical conditions with a well-defined arachnoid plane between nerve and vein comparable to a typical nerve-artery compression. Finally, it was noticed whether an indentation or groove in the trigeminal nerve was seen.

The study was approved by the local ethics board. Informed consent was not obtained from every patient, since all examinations had been on regular basis in the outpatient clinic.

## Demonstration of results and statistical analysis

Immediate clinical outcome after surgery was evaluated. Therefore, postoperatively evaluation of pain was simply graded into complete pain relief, partial improvement or no pain relief. Hypesthesia or dysesthesia right after surgery was recorded. The latest clinical follow-up included the total evaluation score by the Barrow Neurological Institute (BNI) consisting of pain intensity and facial numbness scores [5].

Early clinical evaluation and follow-up BNI score was statistically analyzed regarding type of decompression, types of venous compression (Table 1), neurovascular adherence (Type 1 or 2), caliber of the vein, trigeminal cistern (narrow, wide), indentation/groove (yes or no).

Statistical analysis was conducted with SAS 9.3 (SAS Institute, USA). Fisher’s exact test for categorical variables was conducted, with *p*-values < 0.05 used to define significance.

## Results

### Evaluation of radiological and intraoperatively obtained characteristics

Transposition of the vein with introduction of a small piece of Teflon between nerve and vein was performed in 32, coagulation/division in 17 patients. Medium-sized veins were found in 24 (49%), large veins in 15 (30.6%), and small veins in 10 (20.4%) patients. Neurovascular adherence Type 1 was seen in 30 (61.2%), Type 2 in 19 (38.7%) patients. An indentation of the trigeminal nerve was seen in 7 (14.3%) patients. The types of venous compression described by Inoue et al. [7] were categorized. We found Type A in 12 (24.5%), B in 11 (22.4%), C in 10 (20.4%), and D in 16 (32.7%) patients.

The trigeminal cistern revealed an average size of 1.67 cm^2^ (0.75–3.21 cm^2^) and mean value of the length of the trigeminal nerve of 7.1 mm (3–12). For verification of the data, Pearson’s correlation of the parameters was strongly significant (*p* < 0.001). With respect to this analysis trigeminal cistern was wide in 27 (> 1.67cm^2^) and narrow in 22 patients (Table 3).

**Table 3:** Intraoperatively obtained anatomical/surgical features

Number of patients	49
Trigeminal cistern	
narrow wide	22 27
Type of venous compression by Inoue et al. (2017)^7^	
A B C D	12 11 10 16
Caliber of the vein	
small medium large	10 24 15
Neurovascular adherence	
yes no	30 19
Trigeminal nerve indentation	
Yes No	7 42
Surgical procedure	
coagulation/division transposition (Teflon)	17 32

### Postoperative outcome

Evaluation on discharge revealed complete pain relief in 39 (80%), partial pain improvement in 7 (14%), and no benefit in 3 (6%) patients. One of the patients who did not improve initially, reported pain relief after several months. Facial hypesthesia or paresthesia was reported by 14 (28.6%) patients. None of them reported sensory deficits before surgery. Three of them revealed slight improvement at final FU,11 patients remained unchanged. Whether a change of auditory evoked potentials was observed or not in relation with venous sacrifice could not be analyzed retrospectively.

Postoperative complications were seen in 4 patients. There was a transient partial hearing loss and dizziness in one patient each. In another patient, meningitis was diagnosed and treated with antibiotics. One patient developed transient double vision due to a venous congestion with cerebellar edema. In this patient, a compressive petrosal vein was sacrificed. Fortunately, clinical symptoms and edema resolved completely.

Mean follow-up (FU) was 42.1 months (range: 3–144 months). Two patients reported a resolution of pain after primary unsatisfactory benefit. Two patients never got any benefit from surgery. After a pain free interval, six patients (12.2%) developed recurrent pain within 4 months up to 7 years.

With respect to overall BNI evaluation (Table 4), the BNI pain intensity score revealed 71.4% excellent to very good scores if score 1 and 2 are included (score 1: 32 (65.3%); 2: 3 (6.1%); 3: 6 (12.2%); 4: 4(8.2%), 5: 4(8.2%)). BNI facial numbness score 3 was only present in one patient (2%) but score 2 could be detected in 13 patients (26.5%).

**Table 4:** BNI score on follow-up

BNI pain intensity score	
1 (no pain, no med.)	32
2 (occasional pain, not requiring med.)	3
3 (some pain, adequately controlled with med.)	6
4 (some pain, not adequately controlled with med.)	4
5 (severe pain, no pain relief)	4
BNI facial numbness score	
1 (no facial numbness)	26
2 (mild facial numbness, not bothersome)	13
3 (facial numbness, somewhat bothersome)	1
4 (facial numbness, very bothersome)	0
BNI total (pain + numbness score)	
Excellent (2)	20
Good (3)	14
Fair (4)	5
Poor (≥ 5)	10

We offered 6 patients with recurrent pain (primarily treated with coagulation/division: 1; transposition/Teflon sponge: 5) a re-exploration. All of them were informed about the option of a partial sensory rhizotomy (PSR). Three of them refused this option. In none of the patients, a new neurovascular conflict was found. Three patients received PSR. The Teflon sponge was removed in all patients if feasible (neurolysis). On discharge, patients who received a PSR reported a resolution of pain. The remaining 3 patients reported a slight benefit.

### Statistical analysis

There was no statistical relationship between immediate pain improvement and kind of surgical procedure, size of trigeminal cistern, type of venous compression, venous caliber, trigeminal nerve indentation or neurovascular adherence. BNI pain intensity score on FU was analyzed with all aspects. There was no significant relationship.

There were 14 patients (28.6%) with a BNI facial numbness score of 2 (13 patients) or 3 (1 patient). Statistical analysis revealed a significant value (*p* < 0.05) with respect to the type of venous compression. Type C and D of Inoue et al. [7] presented with a significant relation of BNI facial numbness score. The exact number of Type D revealed 8/16 patients with a facial numbness on FU, Type C 5/10 and Type B 1/11 respectively. The other features did not show any influence on facial numbness. Occurrence of postoperative facial numbness did not significantly differ between the coagulation/division and teflon interposition groups (Fisher’exact test *p* = 0.74)).

Additionally, we did a correlation analysis for pain relief and presence vs absence of facial numbness. The Fisher’s exact test for categorical variables was used. We found no correlation between pain relief versus presence or absence of facial numbness (BNI pain intensity vs facial numbness scores on FU *p* = 0.48).

Further analysis was performed to evaluate differences between atypical and typical TN with respect to FU BNI pain intensity and pain recurrence. There was no significant association between both groups.

## Discussion

Purely venous compression has taken into account as a cause of TN. Several studies report the relevance [2, 3, 6, 7, 11, 18]. Prognostic factors that may predict a successful outcome after MVD are difficult to establish. Arterial compression at TREZ in combination with typical symptoms of TN predicts a good outcome after MVD [1]. In contrast, venous anatomy may be more complex [3, 6, 7, 11]. A neurovascular contact can vary through the course of the nerve. This observation corresponds well with other studies [3, 7, 11]. We therefore decided to classify venous compression regarding the type of Inoue et. al (Table 1) [7]. The endoscope-assisted technique enabled us to get a better overview of the local venous anatomy that is sometimes not clearly visible with the microscope. Especially, the view into Meckel’s cave can easily be done with angulated optics**.** As mentioned by Dumot and Sindou, venous conflicts would be missed if exploration is not systematically done [3]. We definitely agree that exploration should be done from TREZ up to Meckel’s cave.

### Relevance of anatomical and surgery-related characteristics

Statistical analysis excluded any influence of the compression site on postoperative outcome. Whether, the compression was located at TREZ, midline portion or Meckel’s cave made no difference. The hypothesis that venous caliber might rather affect the success of MVD could not be confirmed. Understanding of the venous drainage through the superior petrosal venous system as mentioned by Dumot et al. [2, 3] is of great importance to decide whether to coagulate/divide a compressing vein or to do a trans-/interposition. Sacrificing the superior petrosal vein can carry the risk of venous congestion and hemorrhage [13]. We had one case with a cerebellar edema following coagulation/ division. This example reminded us to be more accurate with the identification of the venous anatomy. Our general strategy is based on preservation of the SPV complex. We coagulate veins if they are small (< 2 mm) or we can clearly identify collateral drainage into the superior petrosal sinus.

One could presume that a complete resolution of a neurovascular conflict by eliminating the venous cause should provide an advantage compared to classic microvascular decompression. But statistical analysis did not show a significant difference between the surgical procedures regarding pain resolution. There was also no relation of the surgical procedure with respect to postoperative facial hypesthesia/dysesthesia or BNI facial numbness score on FU. Altogether, we recognized 14 patients (28.6%) with a facial sensory deficit still present at FU. Only a few studies report an observation of facial numbness of 20.7%, 26.7% or 29% and 22% at FU [3, 6, 7]. Direct manipulation of the trigeminal nerve or coagulation of a vein may be considered as a cause in our opinion and others [6, 11]. However, we found Type C and D venous compression presented with a significant relation to a relevant BNI facial numbness score. We have seen 50% of facial numbness postoperatively for each type. That means, both a large transverse pontine vein (TPV) with distal trigeminal nerve compression close to Meckel’s cave and a venous compression along the entire trigeminal nerve carry a risk of postoperative facial numbness independently from the surgical procedure. Local neurovascular adherence was not related to the BNI score in any form.

### Pain relief and recurrence

Complete pain relief on FU was achieved for 32 (65.3%) patients. Some studies report full pain resolution on FU of 60%, 67%, 69.6%, and 80% respectively [3, 6–8]. A meta-analysis was done by Soni et al. about the outcome with purely venous compression [18]. Out of 19 studies they found a total BNI I (excellent) score of 75.6%. The score was independent from the surgical procedure. Our data support this fact. Pain recurrence rate of 12.2% in our group is little lower compared to the average value of 23.1% of the meta-analysis [18]. Yet, mean FU to pain recurrence is also lower compared to the mentioned study (43 to 51.4 months). In none of the cases we re-explored, newly developed offending vessels were found as reported by others [8, 9].

## Limitations

The main limitations of our study are the small number of patients and the short follow up period. Furthermore, it is a single center evaluation. Venous compression of the trigeminal nerve as a cause of TN is still rare. Factors included in this study, like neurovascular adherence or caliber of the offending veins are based on a subjective evaluation of the authors.

## Conclusion

Doubtless venous compression of the trigeminal nerve is one of the causes of classic TN. Venous anatomy is very variable and complex. It is widely accepted that the offending vein doesn't necessarily have to be located at TREZ but through the entire course of the nerve up to Meckel’s cave [3, 7, 11, 20].

We did not find typical anatomical features that could either predict or influence the outcome regarding pain improvement or resolution in any form. Neither classic microvascular decompression (interpsiton/transposition) nor sacrificing the offending vein made any difference in outcome. Facial numbness is a typical complication after surgery and strongly associated with venous compression type C and D according to Inoue et al. [7].
